# Leukocyte telomere length and attrition in association with disease severity in cystic fibrosis patients

**DOI:** 10.18632/aging.206093

**Published:** 2024-08-29

**Authors:** Dries S. Martens, Elise J. Lammertyn, Pieter C. Goeminne, Kristine Colpaert, Marijke Proesmans, Bart M. Vanaudenaerde, Tim S. Nawrot, Lieven J. Dupont

**Affiliations:** 1 Centre for Environmental Sciences, Hasselt University, Hasselt, Belgium; 2 Department of Chronic Diseases and Metabolism (CHROMETA), Laboratory of Respiratory Diseases and Thoracic Surgery (BREATHE), KU Leuven, Leuven, Belgium; 3 Hospital VITAZ Sint-Niklaas, Sint-Niklaas, Belgium; 4 Department of Respiratory Diseases, University Hospitals Leuven, Leuven, Belgium; 5 Department of Pediatrics, Pediatric Pulmonology, University Hospital of Leuven, Leuven, Flanders, Belgium; 6 Department of Public Health and Primary Care, KU Leuven, Leuven, Belgium

**Keywords:** cystic fibrosis, telomere length, biological aging, lung diseases, patient cohort

## Abstract

Cystic fibrosis (CF) is characterized by chronic airway inflammation and premature aging. The link with leukocyte telomere length (LTL) as a marker of biological aging is unclear. We studied disease severity and LTL in 168 CF patients of which 85 patients had a second retrospective LTL assessment. A higher FEV_1_ was associated with longer LTL, with a stronger effect in men (5.08% longer LTL) compared to women (0.41% longer LTL). A higher FEV_1_/FVC ratio was associated with 7.05% (*P*=0.017) longer LTL in men. CF asthma, as defined by the treatment with inhaled corticosteroids, was associated with -6.65% shorter LTL (*P*=0.028). Men homozygous for the ΔF508 genotype showed a –10.48% (*P*=0.026) shorter LTL compared to heterozygotes. A genotype-specific non-linear association between LTL shortening and chronological age was observed. Stronger age-related LTL shortening was observed in patients homozygous for the ΔF508 genotype (*P*-interaction= 0.044). This work showed that disease severity in CF patients negatively influences LTL, with slightly more pronounced effects in men. The homozygous genotype for ΔF508 may play a role in LTL attrition in CF patients. Understanding factors in CF patients that accelerate biological aging provides insights into mechanisms that can extend the overall life quality in CF-diseased.

## INTRODUCTION

Aging is a complex biological process characterized by the progressive weakening of almost all physiological functions resulting in a time-dependent increase in mortality [1, 2]. The molecular mechanisms behind aging are being explored including cellular senescence [2]. Senescence encompasses a process that imposes permanent proliferative arrest on aged cells that have been chronically accumulating damage over the years until they eventually reach a threshold of cellular stress [3]. Possible inducers of cellular senescence include oxidative stress, DNA damage, and telomere shortening [4]. Telomeres are stretches of repetitive DNA (tandem TTAGGG repeats) that cap the ends of chromosomes, protecting them from unscheduled DNA repair and degradation. In somatic cells, telomeres undergo attrition at each cell replication until they become dysfunctional and trigger a DNA damage response causing cellular senescence [5, 6].

A growing body of research illustrates the association between chronic, age-related respiratory diseases and telomere shortening as it has been shown that patients suffering from chronic asthma, chronic obstructive pulmonary disease (COPD) and idiopathic pulmonary fibrosis (IPF) all demonstrate shorter telomeres than age-matched controls [7–11]. Furthermore, there is evidence for the presence of increased cellular senescence in the cystic fibrosis (CF) airways. Fischer et al. demonstrated an elevated expression of the neutrophil elastase-induced senescence marker p16 and DNA damage response markers in airway epithelial cells of CF patients [12]. However, telomere length (TL) in these cells did not differ when compared with controls, although some CF subjects did exhibit telomere shortening. Furthermore, no difference in TL was observed in CF lung tissue compared with healthy tissue [9] and in leukocytes of CF patients when compared with age-matched controls [13]. In this latter study, comorbidities, a higher number of hospitalization days and inhaled corticosteroids treatment (ICS) were associated with shorter leukocyte telomere length [13].

Based on the European patient registry report, CF patients make up a quite young population with a mean age across all Western European countries of 21.2 years. CF used to be a predominantly pediatric disease with a life expectancy of about 29 years at the beginning of the 1990’s. Currently, 54% of patients reach adulthood and have a median predicted survival age that has risen to 41 years. Therefore, clinicians become increasingly challenged by aging and age-related diseases such as osteopenia and osteoporosis, and malignancies of the gastrointestinal tract [14, 15]. These cancers have a higher frequency in CF patients and occur at a younger age compared to the general population [16].

We hypothesized that CF patients with more severe disease characteristics exhibit shorter leukocyte telomere length (LTL) and greater LTL attrition. This suggests increased cellular senescence, which may contribute to accelerated aging and a higher incidence of age-related diseases. Therefore, we determined LTL and LTL attrition (change in LTL measured at two different timepoints) in our CF population consisting of both adults and children and correlated our measurements with clinical characteristics.

## RESULTS

### Patient characteristics

Demographic and clinical patient characteristics are summarized in Table 1. The study population consisted of 168 CF patients, of which data was collected between 2014 and 2015. Of this population, 30% were below 18 years of age, resulting in a mean age of 23.8 years (range: 4-55) and 47% were females. All patients, except one, were of European descent. Mean FEV_1_ 76.8% predicted (range: 25-151) and mean BMI was 20.8 kg/m^2^ (range: 14-38). In total 42% of included patients were treated with ICS, 14% had a history of smoking of whom 10% used to smoke, 2% actively smoked and 2% were passively exposed to smoke in the household. From 85 patients out of the 168 included patients, a historical DNA sample and data was retrospectively obtained between 1990 and 2013. The data retrospectively collected (n=85) are referred to as timepoint 1 (TP1) and the data collected for the 168 patients between 2014 and 2015 are referred to as timepoint 2 (TP2), see Supplementary Figure 1. Characteristics of this subgroup (n=85) at TP2 did not differ from the overall study group at TP2 (n=85). These patients had a mean age of 9.5 years (range: 0-39) at TP1. The mean follow-up time between TP1 and TP2 was 11.5 years (range: 1.8-23.6).

**Table 1:** Demographic and clinical characteristics of all cystic fibrosis diagnosed patients at timepoint 1 and timepoint 2.

**Characteristic**	**All patients at TP2**	**Subgroup at TP2**	**Subgroup at TP1**
No. (%)	168	85	85
Children <18, No. (%)	51 (30.4%)	36 (42.4%)	64 (75.3%)*
Age, mean (SD), yrs	23.8 (11.2)	20.7 (10.5)	9.5 (10.3)*
BMI, mean (SD), kg/m^2^	20.8 (3.8)	20.5 (3.9)	NA
Sex			
Men, No. (%)	89 (53%)	48 (56.5%)	48 (56.5%)
Women, No. (%)	79 (47%)	37 (43.5%)	37 (43.5%)
FEV_1_, mean (SD), % predicted	76.8 (26.5)	81.1 (23.3)	NA
FVC, mean (SD), % predicted	94.4 (20.3)	96.8 (16.8)	NA
FEV_1_/FVC, mean (SD), ratio	0.68 (0.15)	0.71 (0.15)	NA
LTL, mean (SD), T/S, ratio	0.83 (0.19)	0.83 (0.20)	1.32 (0.36)*
CFTR-genotype			
Homozygous, No. (%)	90 (53.6%)	44 (51.8%)	44 (51.8%)
Heterozygous, No. (%)	78 (46.4%)	41 (48.2%)	41 (48.2%)
European ethnicity, No. (%)^a^	166 (98.8%)	84 (98.8%)	84 (98.8%)
Corticosteroid intake^b^			
None, No. (%)	93 (55.4%)	48 (56.5%)	NA
Oral, No. (%)	5 (2.9%)	2 (2.3%)	NA
Inhaled (ICS), No. (%)	70 (41.7%)	35 (41.2%)	NA
Smoking history			
Never, No. (%)	145 (86.3%)	75 (88.2%)	NA
Active, No. (%)	4 (2.4%)	2 (2.4%)	NA
Former, No. (%)	15 (8.9%)	6 (7.0%)	NA
Passive, No. (%)	4 (2.4%)	2 (2.4%)	NA

Data presented as mean (SD) or as frequency, No. (%). Statistical difference with left adjacent group *P<0.001. Smoking history is defined as never smoking, active smoker, former smoker (patient smoked before TP2 but currently not smoking), passive smoker (patient is exposed to smoke in the household environment). Genotype was defined as homozygous (ΔF508/ΔF508) or heterozygous (ΔF508/other or other/other). Mean (SD) follow-up period for 85 patients between TP1 and TP2 was 11.5 (5.7) years. Abbreviations: TP1, timepoint 1; TP2, timepoint 2; BMI, body-mass index; FEV_1_, forced expiratory volume in 1 second; FVC, forced vital capacity; LTL, leukocyte telomere length; CFTR, Cystic fibrosis transmembrane conductance regulator; ICS, inhaled corticosteroids.

^a^Data missing on n=1.

^b^see supplementary Table 1 for a list of brand names and active substances.

### General demographic determinants of LTL in CF patients

**Figure 1:**
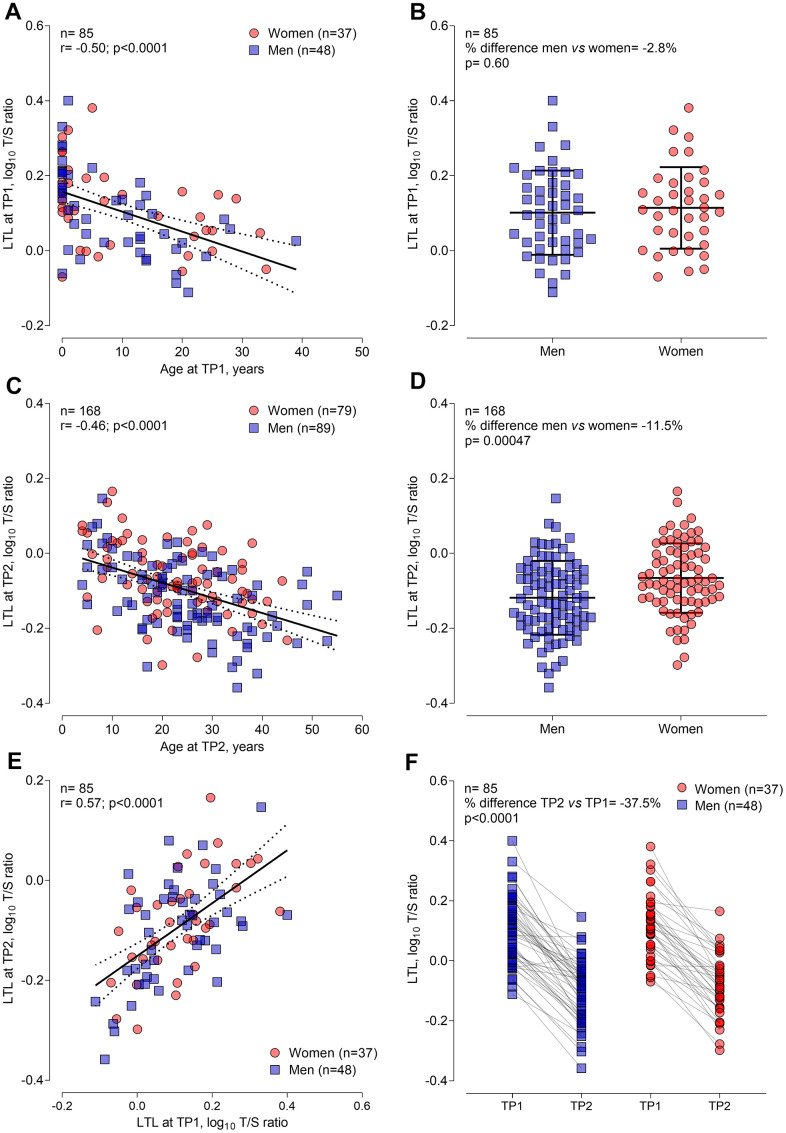
**General demographic determinants of leukocyte telomere length at timepoint 1 (n=85) and at timepoint 2 (n=168) in CF patients.** (**A**) and (**C**) Pearson correlation of LTL with age at TP1 and TP2, respectively. (**B**) and (**D**) Sex differences in LTL at TP1 and TP2, respectively. (**E**) Pearson correlation of LTL at TP1 and TP2, representing LTL tracking over time. (**F**) Decline in LTL for 85 participants from TP1 to TP2. Abbreviations: TP1: timepoint 1; TP2: timepoint 2; LTL: leukocyte telomere length.

LTL decreased with chronological age at TP1 and TP2 (Figure 1A, 1C, respectively). Each year increment was associated with –1.22% (95% CI: –0.74 to –0.33%; *P*<0.001) shorter LTL at TP1, and with –0.93% (95% CI: –1.20 to –0.65%; *P*<0.001) shorter LTL at TP2. At TP1 women did not show a difference in LTL compared with men but showed longer LTL in the study group at TP2 (Figure 1B, 1D, respectively). At TP2 women had 10.5% (95% CI: 4.0 to 17.4%; *P*=0.0015) longer LTL compared to men, after age adjustment. LTL showed a clear tracking in 85 CF patients between TP1 and TP2 as shown by the LTL correlation within the same subjects (r=0.57, *P*<0.001; Figure 1E). LTL ranged from 0.77 to 2.51 in blood samples at TP1 and from 0.44 to 1.46 in samples at TP2, resulting in a significantly lower LTL at TP2 compared with TP1 (*P*<0.001). This difference was comparable for men and women as shown in Figure 1F.

### Association of clinical CF predictors with LTL

In unadjusted and after adjustment for age, sex, BMI, and smoking history, FEV_1_ and the FEV_1_/FVC ratio were positively associated with LTL (Table 2). Each SD increment in FEV_1_ and FEV1/FVC ratio was associated with 4.13% (95%CI: 0.33 to 8.08%; *P*=0.033) and 3.98% (95%CI: –0.02 to 8.14%; *P*=0.051) longer LTL, respectively. These associations were observed in men (6.14%; p=0.023 and 8.46%; *P*=0.0028, longer LTL for each SD increment in FEV_1_ and FEV_1_/FVC ratio, respectively) but not in women (0.76%; *P*=0.79 and –2.74%; *P*=0.36, difference in LTL for each SD increment in FEV_1_ and FEV_1_/FVC ratio, respectively). For the association between FEV1/FVC ratio and LTL a significant sex-specific association was observed (*P*-interaction=0.014). Further adjusting these models for ICS use and genotype, slightly attenuated the effects but were confirmative. FVC was not associated with LTL in CF patients (Table 2). Patients using ICS as part of their daily maintenance therapy (n=70), demonstrated –7.19% (95% CI: –14.41 to –0.43%; *P*=0.037) shorter LTL compared with patients not using ICS (Table 2). Effects were comparable for men and women, and further adjustment for genotype did not alter these results (Table 2). Lastly, genotype, as a surrogate parameter for disease severity, was not associated with LTL. After stratifying for sex, male homozygous CF patients (n=48) had –10.46% (95% CI: –20.66 to –1.12%; *P*=0.028) shorter LTL compared with male heterozygotes (n=41). No association was observed in women, however no interaction (*P*=0.14) between genotype and sex was observed (Table 2). Additionally, adjusting for ICS did not alter these results.

**Table 2:** Association of clinical cystic fibrosis predictors with leukocyte telomere length in cystic fibrosis patients (n=168).

	**Total population (n=168)**		**Men (n=89)**		**Women (n=79)**		***P*-interaction^a^**
	**% difference (95% CI)**	***P*-value**	**% difference (95% CI)**	***P*-value**	**% difference (95% CI)**	***P*-value**	
**FEV_1_**							
Model1	6.89 (3.37, 10.52)	<0.001	9.58 (4.85, 14.54)	<0.001	4.76 (-0.07, 9.83)	0.053	0.11
Model2	4.13 (0.33, 8.08)	0.033	6.14 (0.83, 11.73)	0.023	0.76 (-4.87, 6.74)	0.79	0.18
Model2+ICS and genotype	3.35 (-0.46, 7.29)	0.085	5.08 (-0.13, 10.55)	0.056	0.41 (-5.35, 6.51)	0.89	0.15
**FVC**							
Model1	2.12 (-1.37, 5.74)	0.24	1.57 (-3.21, 6.58)	0.52	4.27 (-0.57, 9.34)	0.084	0.53
Model2	2.10 (-1.25, 5.57)	0.22	0.91 (-3.73, 5.76)	0.70	2.11 (-3.05, 7.54)	0.43	0.52
Model2+ICS and genotype	2.07 (-1.26, 5.50)	0.22	1.16 (-3.40, 5.94)	0.62	2.03 (-3.18, 7.52)	0.45	0.76
**FEV_1_/FVC**							
Model1	8.83 (5.35, 12.42)	<0.001	12.62 (8.08, 17.35)	<0.001	3.17 (-1.65, 8.24)	0.20	0.011
Model2	3.98 (-0.02, 8.14)	0.051	8.46 (2.93, 14.28)	0.0028	-2.74 (-8.41, 3.29)	0.36	0.014
Model2+ICS and genotype	2.64 (-1.47, 6.92)	0.21	7.05 (1.28, 13.16)	0.017	-3.35 (-9.10, 2.76)	0.27	0.017
**ICS^b^**							
Model1	-14.21 (-22.21, -6.73)	<0.001	-14.29 (-25.31, -4.24)	0.0050	-11.49 (-22.81, -1.21)	0.028	0.71
Model2	-7.19 (-14.41, -0.43)	0.037	-7.05 (-16.93, 2.00)	0.13	-6.81 (-18.03, 3.34)	0.19	0.89
Model2+genotype	-7.56 (-14.80, -0.78)	0.028	-7.07 (-16.71, 1.77)	0.12	-6.56 (-17.96, 3.74)	0.22	0.96
**Genotype^c^**							
Model1	1.00 (-6.17, 7.68)	0.78	-1.21 (-11.45, 8.08)	0.80	3.62 (-6.10, 12.44)	0.45	0.48
Model2	-4.46 (-11.37, 2.01)	0.18	-10.46 (-20.66, -1.12)	0.028	2.37 (-7.37, 11.23)	0.62	0.14
Model2+ICS	-4.98 (-11.85, 1.47)	0.13	-10.48 (-20.59, -1.22)	0.026	1.50 (-8.40, 10.50)	0.75	0.17

Estimates presented as %difference in average relative LTL for an SD increment in FEV_1_ (+26.5% predicted for total, +25.2% for men, +28.1% for women), FVC (+20.3% predicted for total, +18.5% for men, +21.9% for women), FEV_1_/FVC (+0.15 for total, +0.16 for men, +0.14 for women) or for the use of ICS vs. non-users and the homozygous vs. heterozygous genotype. Abbreviations: FEV_1_, forced expiratory volume in 1 second; FVC, forced vital capacity; ICS, inhaled corticosteorids.

Model 1: unadjusted model.

Model 2: adjusted model for age, sex, BMI, and smoking history.

^a^P-interaction for sex*predictor effect.

^b^n=70 for ICS use of which n=29 women and n=41 men.

^c^n=90 homozygous for the ΔF508 genotype of which n=42 women and n=48 men.

### Predictors of leukocyte telomere attrition in CF patients

**Figure 2:**
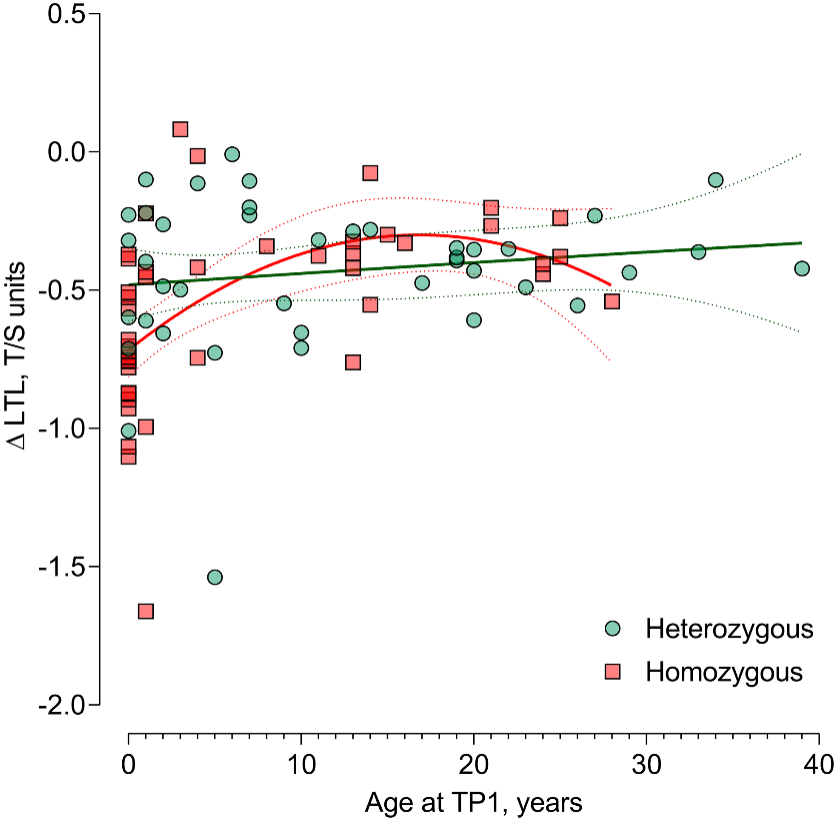
**Difference in age-dependent leukocyte telomere length change from timepoint 1 to timepoint 2 in ΔF508 homozygous vs. heterozygous.** Non-linear (quadratic) association of age with the LTL attrition in CF patients homozygous for the ΔF508 mutation (n=44) compared with heterozygous patients (n=41) P-interaction between the quadratic term of age at TP1 and genotype (*P*=0.044), reflecting the genotype-specific non-linear LTL attrition-age association. Abbreviations: TP1: timepoint 1; TP2: timepoint 2; LTL: leukocyte telomere length.

In 85 CF patients, telomere attrition could be evaluated. Figure 2 shows the unadjusted association between age at TP1 as a predictor for LTL attrition (ΔLTL). We observed a non-linear (quadratic) age effect on LTL attrition with stronger attrition at younger ages. This was significantly different according to genotype. After adjusting for sex and time between TP1 and TP2, this non-linear age association with LTL attrition remained different according to genotype (*P*-interaction=0.044) (Table 3). In CF patients homozygous for the ΔF508 mutation, the LTL attrition was highest in very young children and declined throughout childhood and adolescence. At the age of 1, each year increment in age showed less strong attrition of 0.052 T/S ratio (95%CI: 0.023 to 0.081; *P*<0.001) in homozygous CF patients. This effect was lowered at the age of 5 years to 0.036 T/S ratio (95%CI: 0.018 to 0.055; *P*=0.0021) and was not observed at the age of 17 (Table 3). For patients heterozygous for the ΔF508 mutation, no significant association between age and LTL attrition was observed (Table 3). In models correcting for the regression of the mean phenomenon, these results were confirmed (Supplementary Table 2).

**Table 3:** Genotype-specific non-linear age association with leukocyte telomere length attrition in cystic fibrosis patients (n=85).

**Genotype**	**Age**	**β (95% CI)**	**p-value**
Homozygous	1	0.052 (0.023, 0.081)	<0.001
Heterozygous	1	0.009 (–0.014, 0.032)	0.43
Homozygous	5	0.036 (0.018, 0.055)	0.0021
Heterozygous	5	0.007 (–0.010, 0.024)	0.43
Homozygous	17	–0.006 (–0.022, 0.012)	0.52
Heterozygous	17	0.000 (–0.008, 0.008)	0.97

Estimates presented with 95%CI representing the association between ΔLTL (LTL at TP2 minus LTL at TP1, in T/S ratio) and age at TP1 for each year increment at different ages (1, 5, and 17 years, based on 25^th^, median, and 75^th^ percentile of the age distribution). Models adjusted for sex and time between samples within an individual. P-interaction between the quadratic term of age at TP1 and genotype (p=0.044), reflecting the genotype-specific non-linear LTL attrition-age association. In total 44 patients were homozygous for the ΔF508 mutation and 41 were heterozygous.

## DISCUSSION

The present study demonstrated that CF patients suffering from more severe lung disease, homozygous for the ΔF508 mutation, or diagnosed with CF asthma (as defined by the use of ICS), have shorter LTL. The associations of FEV_1_, FEV_1_/FVC ratio, and genotype with shorter LTL were potentially sex-specific. Furthermore, there was a non-linear effect of age on the LTL attrition which was significantly modified by genotype. Homozygous patients demonstrated a stronger LTL attrition during childhood, decreasing into adolescence, steady during adulthood, and eventually increasing again at an older age. In contrast, heterozygous patients showed a less strong association between LTL attrition and age, resulting in steady LTL shortening throughout life.

Our findings on the age-dependent LTL attrition in homozygous patients are in line with previous findings of rapid LTL attrition during the first 20 years of life [17], resulting in a virtually fixed ranking of an individual’s LTL compared to the general population for the rest of the adult life [18, 19]. We have demonstrated a positive intra-individual correlation between LTL at the two different evaluated timepoints l, indicating that patients having long or short LTL at a given timepoint will still have a long or short LTL at a later timepoint in life, respectively. Taken together, our data suggest that the ΔF508 homozygous genotype, resulting in the full absence of cystic fibrosis transmembrane conductance regulator (CFTR) channels on the cell surface and therefore giving rise to a severe phenotype [20], exerts its effect on LTL attrition already early in life and maybe even already intra-uterine, resulting in shorter LTL remaining anchored for the rest of the adult life.

These observations on telomere biology in CF patients may have important clinical implications. Early diagnosis, preferably with genetic analysis, and rapid start-up of appropriate therapy is key for the evolution of the disease in later life. Widespread implementation of systematic newborn screening for CF may lead to faster diagnosis. Recently, two phase 3 clinical trials have been reported during which a combination of small molecule therapy targeting the CFTR protein dysfunction under the form of ivacaftor, a CFTR potentiator, and lumacaftor, a CFTR corrector was administered to patients 12 years of age or older homozygous for the ΔF508 mutation [21]. Compared with placebo, this combination improved FEV_1_ and reduced the frequency of pulmonary exacerbations. Our findings suggest that small molecule therapy may also be beneficial to younger children as it may partly restore CFTR function possibly leading to a reduced LTL attrition.

Female CF patients appeared to be less sensitive to the associations of FEV_1_ and genotype with shorter LTL. A possible explanation could be that women in general have intrinsically longer telomeres compared with men [22], which may indicate a higher protective capacity against telomere-influencing factors and therefore may reduce their effect. Two potential mechanisms are described which may contribute to longer LTL in females. Firstly, the female sex hormone estrogen diminishes oxidative stress, which is known to induce telomere shortening [23]. Besides, estrogen stimulates the transcription of the gene encoding for telomerase. Telomerase maintains TL in gametes and stem cells by adding guanine-rich repetitive sequences [24, 25]. Subsequently, stem cells give rise to highly proliferative cell populations including blood cells, explaining the longer LTL compared with men. In our study, LTL attrition is especially apparent in pre-pubertal children and progresses more steadily during adulthood, suggesting that the influence of sex hormones such as estrogen on this process is limited. This may explain the lack of a gender-specific genotype-modified effect of age on LTL attrition. Secondly, Skewed X inactivation is a process during which the inactivation of the second X chromosome does not occur randomly, but after the selection of the parental X chromosome that provides a survival advantage [23]. As there is evidence that gene variance on the X chromosome strongly influences TL [26], the X chromosome giving rise to cells with longer telomeres will be selected as they might produce greater tissue reserves. Taken together, these mechanisms may protect female CF patients against the consequences of shorter LTL associated with lower FEV_1_ and the homozygous genotype.

CF patients using ICS demonstrated significantly shorter LTL compared with patients who were not treated with ICS. These observations are in line with recent findings in an independent smaller study of CF-patients [13]. The use of ICS originated during the 1970’s as an alternative to oral corticosteroids for the treatment of asthma and they were soon after prescribed as an anti-inflammatory therapy to CF patients as well, in order to reduce bronchial hyperresponsiveness, wheezing, chronic cough, and bronchospasms. Although the indication of ICS in CF is not straightforward, it is estimated that about 19% of CF patients present with clear symptoms of airway reactivity and are therefore diagnosed with CF asthma [27]. In this study, 41% of patients were prescribed ICS because of the presence of at least an asthmatic component worsening their lung disease. Recently, Belsky et al. demonstrated that patients suffering from chronic, life-course-persistent asthma had shorter LTL compared with gender- and age-matched healthy individuals [8]. Our data suggest that a diagnosis of CF asthma or an asthmatic component complicating the CF lung pathology is associated with increased cellular senescence in CF patients.

Our study has several limitations. First, we did not dispose of DNA of matched healthy individuals, so we could not compare LTL and LTL attrition between CF patients and the general population. In this regard, previous studies did not observe differences in lung TL or leukocyte TL measured in CF patients and healthy controls [9, 13]. In the current study, our aim was to evaluate the link between LTL as a body-wide marker of biological aging in relation to disease severity in a specific CF patient cohort. Intra-individual differences in TL between leukocytes and lung tissue exists due to proliferative differences, nevertheless, TL displays a high intra-individual synchrony and correlation across somatic cells such as leukocytes, lung cells, muscle cells, and fat cells [28, 29]. The latter motivates the idea that leukocyte TL may serve as a body-wide circulatory marker of biological aging in humans, and it has indeed been shown that average relative LTL is causally related to lifespan and age-related diseases [30]. Nevertheless, we acknowledge that tissue-specific and chromosome-specific differences in TL may exert specific disease phenotypes, which was beyond the scope of the current analysis [31]. Second, our sample size of patients having a DNA sample at both evaluated timepoints is limited, and therefore our findings on longitudinal changes in LTL should be interpreted with caution. Nevertheless, this is however inherent to the very specific patient population and the sometimes-wide window of time between both samples. Nevertheless, these are unique samples with equal gender distribution and a homogeneous age range, as is the case for samples collected at TP2. Third, our population included patients from all ages, and developmental trajectories across childhood and adolescence may be different from adults that might have an impact on the observed associations. However, our study is limited in sample size to further evaluated these developmental differences in e.g. age subgroup analyses. Finally, due to unavailability of the sometimes very old patient files, we were not able to retrieve other clinical patient characteristics at TP1, implicating that we were not able to study additional effects on LTL attrition.

## CONCLUSIONS

In CF patients, characteristics of more severe disease were associated with LTL, possibly resulting in an increased rate of aging affecting their other bodily functions and making them more prone to age-related diseases. These effects are probably already established during childhood, stressing the need for early diagnosis of CF and appropriate therapy from an early age onwards.

## MATERIALS AND METHODS

### Study population

176 CF patients (120 adults and 56 children and adolescents younger than 18 years) were included in this study. They were recruited at the University Hospitals Leuven outpatient clinic between April 2014 and September 2015. This recruitment moment is referred to as timepoint 2 (TP2). Written informed consent or written informed parental consent (and written informed assent in case of a minor above the age of twelve) was obtained for all participants. Because of missing genotype determination (n=3) and FEV_1_ (n=5), the final sample size consisted of 168 CF patients (117 adults and 51 children aged under 18). Next, with the consent of participants, we consulted the Centre for Human Genetics of the KU Leuven to explore the availability of existing DNA samples extracted at the time of genotype determination of our study participants. This second, historical DNA sample was available for 107 of the 176 participants (68 adults and 39 children). However, due to improper DNA quality, 22 samples were discarded, resulting in 85 patients with a historical DNA sample (36 adults and 49 children). The moment of historical DNA extraction (between April 1990 and May 2013) is referred to as timepoint 1 (TP1). Supplementary Figure 1 shows a comprehensive timeline and flow chart of the included participants. Clinical characteristics were collected via the electronic patient files. This study was approved by the local ethics committee and the University Hospitals Leuven biobanking centre (S56319).

### Spirometry

Lung function tests were performed according to current American Thoracic Society (ATS)/European Respiratory Society (ERS) recommendations [32, 33]. Because absolute forced vital capacity (FVC) and forced expiratory volume in 1 second (FEV1) expressed in liters are highly dependent on age, and considering the wide age range of patients at TP2, FVC, and FEV1 expressed as the percentage of the predicted value and calculated according to the 2005 ATS/ERS guidelines, was used to assess the effect of lung function on LTL.

### DNA extraction and sample processing

Peripheral blood was collected using 10 ml EDTA vacutainers. After ten minutes of centrifugation at 370 rcf, plasma was removed and the remaining buffy coat was isolated. Buffy coat DNA was extracted using the QIAamp DNA mini kit (Qiagen Inc., Venlo, The Netherlands). DNA concentration and purity were determined using NanoDrop (Thermo Scientific NanoDrop Technologies, Wilmington, DE, USA). DNA integrity was assessed using 1.5% agarose gel electrophoresis to evaluate potential DNA degradation. In total, 22 DNA samples from TP1 showed severe DNA degradation, as evidenced by DNA smears on gels and no intense band of intact DNA, and therefore were excluded.

### CF genotyping

Patients were screened for the ∆F508 mutation in the CFTR gene using the pan-European cystic fibrosis Elucigene CF-EU2v1 kit (Elucigene Diagnostics, Manchester, United Kingdom). This kit detects point mutations, insertions or deletions in the CFTR gene, using a method based on fluorescent amplification refractory mutation system (ARMS). The Elucigene CF-EU2v1 kit can detect the Tn and the TGn polymorphisms and the 50 most common mutations found across the European population, including the ∆F508 mutation. Details on other mutations are provided in Supplementary Material 1. The kit is a multiplexed test which includes two polymerase chain reactions (PCR), A and B. In PCR A the mutant sequences will be detected, together with the Tn and TGn polymorphisms and the wild type sequence for the most common CFTR mutation in Caucasians, ∆F508. In PCR B the corresponding wild type sequences, with exception of the ∆F508 wild type, will be detected. Both PCR reactions include the same non-CFTR internal amplification control markers. DNA was amplified with the following program: 94° C for 20 minutes, followed by 30 cycles consisting of denaturation at 94° C for 1 minute, primer annealing at 58° C for 2 minutes and extension at 72° C for 1 minute. At the end of the program, an additional extension stage at 72° C lasting 20 minutes was included. PCR products were carried out by capillary electrophoresis on the ABI 3730xl Genetic Analyzer (Thermo Fisher Scientific Inc., Waltham, MA, USA) and analysis of the data was performed in the GeneMapper 5 (Thermo Fisher Scientific Inc.) software.

### Leukocyte telomere length measurement

Average relative LTL was measured in triplicate for each sample using a modified qPCR protocol as described previously [34] and in the Supplementary Material 2. All telomeres were measured in triplicate on a 7900HT Fast Real-Time PCR System (Applied Biosystems, Lennik, Belgium) in a 384-well format. Patients that had 2 DNA samples at the 2 timepoints were matched on the same qPCR plate. A 6-point serial dilution of pooled buffy-coat DNA was included to assess PCR efficiency and six inter-run calibrators were included to account for inter-run variability. Relative average LTLs were expressed as the ratio of telomere copy number to single-copy gene number (T/S ratio) relative to the average T/S ratio of the entire sample set (n=261) using the qBase software (for mathematical details we refer to Hellemans [35]). The precision of the assay is evaluated using an intra- and inter-assay intraclass correlation coefficient (ICC). The intra-assay ICC was calculated using all (n=253) triplicate LTL measures (ICC = 0.90; 95%CI: 0.88 to 0.92) and the inter-assay ICC was calculated for a set of samples (n=10) that were analyzed twice within a 1-week interval (ICC = 0.93; 95%CI: 0.71 to 0.98).

### Statistical analysis

All statistical analyses were performed using the SAS 9.4 statistical software (SAS Institute Inc., Cary, NC, USA). LTLs were log_10_ transformed to improve normality. Pearson correlation and student’s t-test were performed to evaluate the association between general demographic determinants and predictors of LTL at both timepoints. Using multivariable-adjusted linear models, clinical disease severity characteristics of CF patients in relation to LTL at TP2 were evaluated. First, the association between FEV_1_, FVC, the FEV_1_/FVC ratio, and LTL was evaluated. Second, the association between the use of ICS (classified as yes or no) and LTL was evaluated. Third, we evaluated whether genotype (homozygous for the ∆F508 mutation or heterozygous) was associated with LTL. All models were adjusted for age, sex, BMI, and smoking history. In additional models, we adjusted the spirometry models for ICS use and genotype. The ICS model was additionally adjusted for genotype and the genotype model for ICS use. As a secondary analysis, all models were stratified for sex and the predictor*sex interaction was evaluated. Estimates are presented as %difference with 95%CI in LTL for a standard deviation (SD) increment in FEV_1_, FVC, FEV_1_/FVC, or the use of ICS vs. non-users and the homozygous vs. heterozygous genotype. Finally, in 85 CF patients with two LTL assessments, telomere attrition was studied. LTL attrition was calculated by the difference in LTL between TP2 and TP1 (ΔLTL=LTL_TP2_–LTL_TP1_). We assessed the non-linear effect of age (modeled as a quadratic term) at TP1 on telomere attrition. Genotype-specific differences were evaluated by including an interaction between genotype and the quadratic age term. Models were adjusted for sex and timing between TP1 and TP2. As a sensitivity analysis, we evaluated whether the results on telomere attrition were robust by considering the potential regression to the mean effect. This was done by replacing ΔLTL with the D score proposed by Verhulst and colleagues [36]. This D score adjusts the difference of consecutive TL measurements by subtracting the change expected from the regression to the mean effect.

## Supplementary Material

Supplementary Materials

Supplementary Figure 1

Supplementary Tables
