# Supplementary material for: Leukocyte telomere length and attrition in association with disease severity in cystic fibrosis patients
Source: Aging (Albany NY). 2024 Aug 29;16(16):11809–23. doi: 10.18632/aging.206093 (PMC11386922; doi:10.18632/aging.206093)
Supplement: Supplementary Materials [file aging-16-206093-s001.pdf]

SUPPLEMENTARY MATERIALS

Supplementary Material 1. Overview of screened mutations in the Elucigene CF-EU2v1 kit

The Elucigene CF-EU2v1 kit can detect the Tn and the TGn polymorphisms and the 50 most common mutations found across the European population: CFTRdele2,3 (21kb), E60X, P67L, G85E, 394delTT, 444delA, R117C, R117H, Y122X, 621+1G>T, 711+1G>T, L206W, 1078delT, R334W, R347P, R347H, A455E, I507del, ΔF508, 1677delTA, V520F, 1717-1G>A, G542X, S549R(T>G), S549N, G551D, R553X, R560T, 1811+1.6kbA>G, 1898+1G>A, 2143delT, 2184delA, 2347delG, W846X, 2789+5G>A, Q890X, 3120+1G>A, 3272-26A>G, R1066C, Y1092X(C>A), M1101K, D1152H, R1158X, R1162X, 3659delC, 3849+10kbC>T, S1251N, 3905insT, W1282X, N1303K. The mutations are described using the established traditional nomenclature.

Supplementary Material 2. Leukocyte telomere length assay

The quantification cycle (C_q) of the telomeric region and the C_q of a single-copy gene (*36B4)* was assessed via a telomeric and single-copy gene specific qPCR respectively. All reactions were run on a 7900HT Fast Real-Time PCR System (Applied Biosystems, Lennik, Belgium) in a 384-well format. A 6-point serial dilution of pooled buffy-coat DNA was included to assess PCR efficiency and six inter-run calibrators were included to account for inter-run variability. C_q-values of the telomere-specific region were normalized relative to the single-copy gene using qBase software (Biogazelle, Zwijnaarde, Belgium). Relative average telomere lengths were expressed as the ratio of telomere copy number to single-copy gene number (T/S ratio) relative to the average T/S ratio of the entire sample set. In qBase, TL is calculated as a calibrated normalised relative quantity (CNRQ) [29]. The latter is achieved by first calculating the RQ based on the delta-Cq method for T and S obtained Cq values, using target specific amplification efficiencies. As the choice of a calibrator sample (sample to which subsequent normalisation is performed) strongly influences the error on the final relative quantities (as a result of the measurement error on the calibrator sample), normalisation is performed to the arithmetic mean quantification values for all analysed samples per cohort, which results in the NRQ. Finally, as samples are measured over multiple qPCR plates, 6 inter-run calibrators (IRCs) are used to calculate an additional correction factor to eliminate run-to-run differences, resulting into the final T/S ratio (CNRQ). Mathematical calculation formulas to obtain RQ, NRQ, and CNRQs are provided by Hellemans [1]. The precision of the assay is evaluated using an intra- and inter-assay intraclass correlation coefficient (ICC). The intra-assay ICC was calculated using all (n=253) triplicate LTL measures (ICC = 0.90; 95%CI: 0.88 to 0.92) and the inter-assay ICC was calculated for a set of samples (n=10) that were analyzed twice within a 1-week interval (ICC= 0.93; 95%CI: 0.71 to 0.98).

The forward and reverse primers used for the qPCR of the telomere-specific region were 5'-ACACTAAGGTTTGGGTTTGGGTTTGGGTTTGGG TTAGTGT-3' and 5'-TGTTAGGTATCCCTATCCC TATCCCTATCCCTATCCCTAACA-3', respectively. The forward and reverse primers for the single-copy gene were 5'GGAATGTGGGCTTTGTGTTC-3' and 5'-CCCAATTGTCCCCTTACCTT-3' respectively. Telomere reaction mixture contained Fast SYBR® Green I dye 2x (Applied Biosystems) master mix, 100 nM forward and 900 nM reverse telomere primer and 12.5 ng DNA. 10 µl of the single-copy gene PCR reaction mixture contained Fast SYBR® Green I dye 2x master mix, forward (10 µM) and reverse (10 µM) primer and 12.5 µg DNA. The thermal cycling profile for the qPCR reaction was as follows: 95° C for 20 seconds, followed by 2 cycles at 94° C for 15 seconds and 49° C for 15 seconds and 40 cycles at 94° C for 15 seconds, 62° C for 10 seconds, and 74° C for 15 seconds.

REFERENCES

1. Hellemans J, Mortier G, De Paepe A, Speleman F, Vandesompele J. qBase relative quantification framework and software for management and automated analysis of real-time quantitative PCR data. Genome Biol. 2007; 8:R19. https://doi.org/10.1186/gb-2007-8-2-r19 PMID:17291332

2. Verhulst S, Aviv A, Benetos A, Berenson GS, Kark JD. Do leukocyte telomere length dynamics depend on baseline telomere length? An analysis that corrects for 'regression to the mean'. Eur J Epidemiol. 2013; 28:859–66. https://doi.org/10.1007/s10654-013-9845-4 PMID:23990212
