# Supplementary material for: Leukocyte telomere length and attrition in association with disease severity in cystic fibrosis patients
Source: Aging (Albany NY). 2024 Aug 29;16(16):11809–23. doi: 10.18632/aging.206093 (PMC11386922; doi:10.18632/aging.206093)
Supplement: Supplementary Figure 1 [file aging-16-206093-s002.pdf]

SUPPLEMENTARY FIGURE

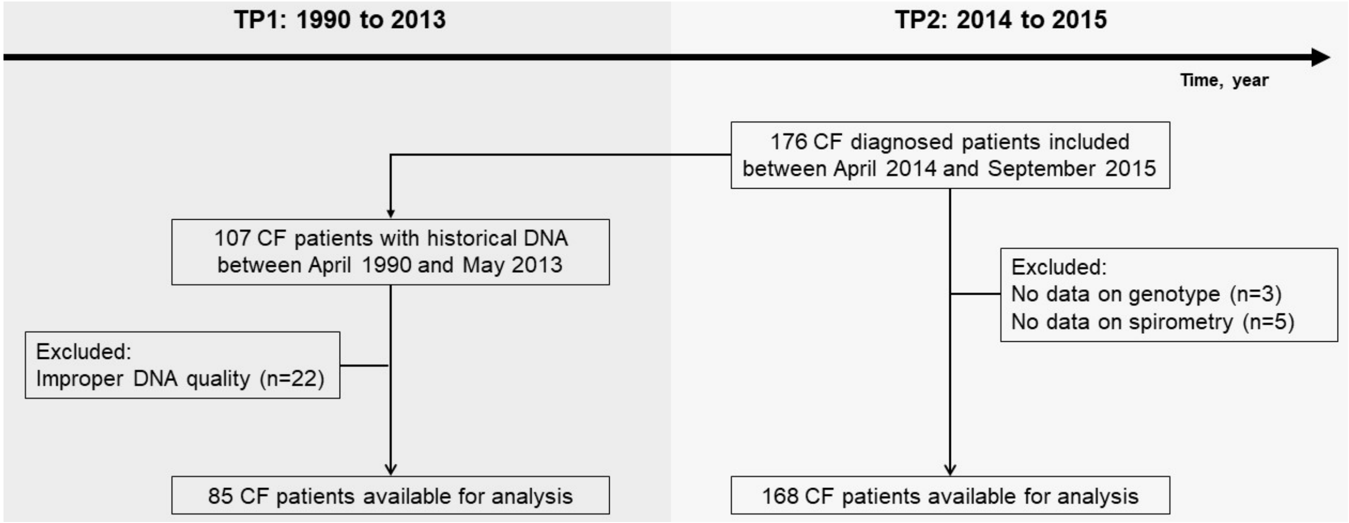

Supplementary Figure 1. Comprehensive time-line with participant selection. In total 176 CF patients were included between April 2014 and September 2015 (timepoint 2, TP2). Due to missing data a final sample size of 168 patients with qualitative TL measurements were available at TP2. A historical DNA sample and a qualitative TL measurement from a subgroup of patients (n=85), participating at TP2, were available between April 1990 and May 2013 (timepoint 1, TP1).
