# Supplementary material for: Leukocyte telomere length and attrition in association with disease severity in cystic fibrosis patients
Source: Aging (Albany NY). 2024 Aug 29;16(16):11809–23. doi: 10.18632/aging.206093 (PMC11386922; doi:10.18632/aging.206093)
Supplement: Supplementary Tables [file aging-16-206093-s003.pdf]

SUPPLEMENTARY TABLES

Supplementary Table 1. List of active substances administered as inhaled corticosteroids (ICS).

Active substance(s)	No. (%)
Budesonide (ICS)/Formoterol fumarate dihydrate (LABA)	39 (55%)
Fluticasone propionate (ICS)/Salmeterol (LABA)	22 (31%)
Fluticasone propionate (ICS)	9 (13%)
Beclometasone dipropionate (ICS) /formoterol fumarate dihydrate (LABA)	1 (1%)

In total, 70 out of 168 included patients were prescribed ICS. In most cases, it concerned a drug combining a long-acting bronchodilator (LABA) and an inhaled corticosteroid (ICS).

Supplementary Table 2. Genotype-specific non-linear age association with LTL attrition in CF patients (n=85) correction for regression to the mean.

Genotype	Age	β (95% CI)	*P*-value
Homozygous	1	0.022 (0.005, 0.039)	0.011
Heterozygous	1	0.001 (–0.013, 0.014)	0.93
Homozygous	5	0.013 (0.002, 0.024)	0.018
Heterozygous	5	–0.001 (–0.011, 0.009)	0.89
Homozygous	17	–0.012 (–0.022, –0.002)	0.021
Heterozygous	17	–0.004 (–0.009, 0.001)	0.084

Estimates presented with 95%CI representing the association between ΔLTL (with correction for regression to the mean as postulated by Verhulst and colleagues [2]) and age at TP1 for each year increment at different ages (1, 5, and 17 years, based on 25th, median and 75th percentile of the age distribution). Models adjusted for sex and time between samples within an individual. P-interaction between the quadratic term of age at TP1 and genotype (*P*=0.039), reflecting the genotype-specific non-linear LTL attrition-age association. In total 44 patients were homozygous for the ΔF508 mutation and 41 were heterozygous.
